# Phage SEP1 hijacks *Staphylococcus epidermidis* stationary cells’ metabolism to replicate

**DOI:** 10.1128/msystems.00263-24

**Published:** 2024-06-21

**Authors:** Maria Daniela Silva, Graça Pinto, Angela França, Joana Azeredo, Luís D. R. Melo

**Affiliations:** 1 CEB-Centre of Biological Engineering, University of Minho, Braga, Portugal; 2 LABBELS–Associate Laboratory, Braga/Guimarães, Portugal; University of Birmingham, Birmingham, United Kingdom

**Keywords:** phage, stationary cells, phage-host interaction, RNA-seq

## Abstract

In nature, bacteria often survive in a stationary state with low metabolic activity. Phages use the metabolic machinery of the host cell to replicate, and, therefore, their efficacy against non-dividing cells is usually limited. Nevertheless, it was previously shown that the *Staphylococcus epidermidis* phage SEP1 has the remarkable capacity to actively replicate in stationary-phase cells, reducing their numbers. Here, we studied for the first time the transcriptomic profiles of both exponential and stationary cells infected with SEP1 phage using RNA-seq to gain a better understanding of this rare phenomenon. We showed that SEP1 successfully takes over the transcriptional apparatus of both exponential and stationary cells. Infection was, however, delayed in stationary cells, with genes within the *gp142-gp154* module putatively implicated in host takeover. *S. epidermidis* responded to SEP1 infection by upregulating three genes involved in a DNA modification system, with this being observed already 5 min after infection in exponential cells and later in stationary cells. In stationary cells, a significant number of genes involved in translation and RNA metabolic and biosynthetic processes were upregulated after 15 and 30 min of SEP1 infection in comparison with the uninfected control, showing that SEP1 activates metabolic and biosynthetic pathways necessary to its successful replication.

**IMPORTANCE:**

Most phage-host interaction studies are performed with exponentially growing cells. However, this cell state is not representative of what happens in natural environments. Additionally, most phages fail to replicate in stationary cells. The *Staphylococcus epidermidis* phage SEP1 is one of the few phages reported to date to be able to infect stationary cells. Here, we unveiled the interaction of SEP1 with its host in both exponential and stationary states of growth at the transcriptomic level. The findings of this study provide valuable insights for a better implementation of phage therapy since phages able to infect stationary cells could be more efficient in the treatment of recalcitrant infections.

## INTRODUCTION

Bacteriophages (phages), the viruses that specifically infect and kill bacteria, are ubiquitous in the environment. Most of our understanding of their interaction dynamics with their host bacteria comes from experiments performed with exponentially growing cells. In natural environments, however, this is not the primary pattern of growth, with bacteria often surviving in the stationary phase (1). As a consequence of starvation, when nutrients are exhausted, these cells are typically in a slower or non-dividing state with low metabolic activity (2). As phages use the metabolic apparatus of the host bacterial cell to replicate, they are generally ineffective against non-growing cells (3). Stationary phase cells are physiologically different than their exponential counterparts, with denser cell envelopes and fewer adsorption sites available, impairing phage infection. Moreover, some phages are reported to enter a “pseudo lysogeny state” or “hibernation mode,” where the phage suspends its replication within the host until more favorable growth conditions are available (4, 5). Still, some phages can have the rare ability to multiply in stationary phase cells. Previously, we showed that the *Staphylococcus epidermidis* phage SEP1 has this unique characteristic, significantly decreasing the number of 48-h-old stationary cells (6). Flow cytometry and quantitative RT-PCR showed that cells responded to phage rapidly after its addition and that active replication of phage genes occurred. More recently, Maffei et al. (7) reported a new *Pseudomonas aeruginosa* phage, named Paride, which can replicate and lyse stationary cells in a deep dormant state (48 h culturing) (7). Controversially, phage T7, previously reported to be active against stationary cells (8), was shown to replicate only on 8-h-old stationary cells, being ineffective against cells cultured for 48 h (7). Despite these important reports, to date, no study investigated the transcriptomic response of stationary cultures exposed to a phage and compared it to exponential-infected cultures. Here, using RNA-seq, we performed a transcriptomic analysis of both exponential and stationary cultures infected with SEP1, aiming to enlighten how SEP1 takes over the transcriptional machinery of the host cell and how the host cell responds to this infection. We showed that SEP1 was able to activate numerous metabolic and biosynthetic processes crucial to the completion of its lifecycle.

## RESULTS AND DISCUSSION

### SEP1 transcription in exponential and stationary cells

Exponential and stationary cultures of *S. epidermidis* were infected with SEP1 phage at a multiplicity of infection (MOI) of 5 to ensure a synchronous infection. RNA was extracted from samples collected shortly before (0 min) and at 5, 15, and 30 min post-infection. These sampling time points were based on the one-step growth curves of SEP1 in exponential and stationary cells (6). On exponential cells, SEP1 had a latency period of 45 min, while on stationary cells the latency period was 75 min. For a comprehensive understanding of gene expression during the phage infection period, it is advisable to perform RNA-seq at multiple time points throughout the latency period. Since the latency period of SEP1 on exponential cells was 45 min (6), we decided to take the samples at multiple time points before this period (5, 15, and 30 min). Our previous study indicated that stationary cells responded quickly after phage addition and that an active replication of phage genes occurred at these early time points. Therefore, in stationary cells, the same time points were considered to try to understand how the phage was taking over the transcriptional resources of the host in this cell state and how the cells responded to the phage infection at these early stages. A more comprehensive time course spanning the entire infection period in stationary cells would provide valuable insights, but practical constraints such as cost and data analysis complexity limited our ability to include additional time points.

**Fig 1:**
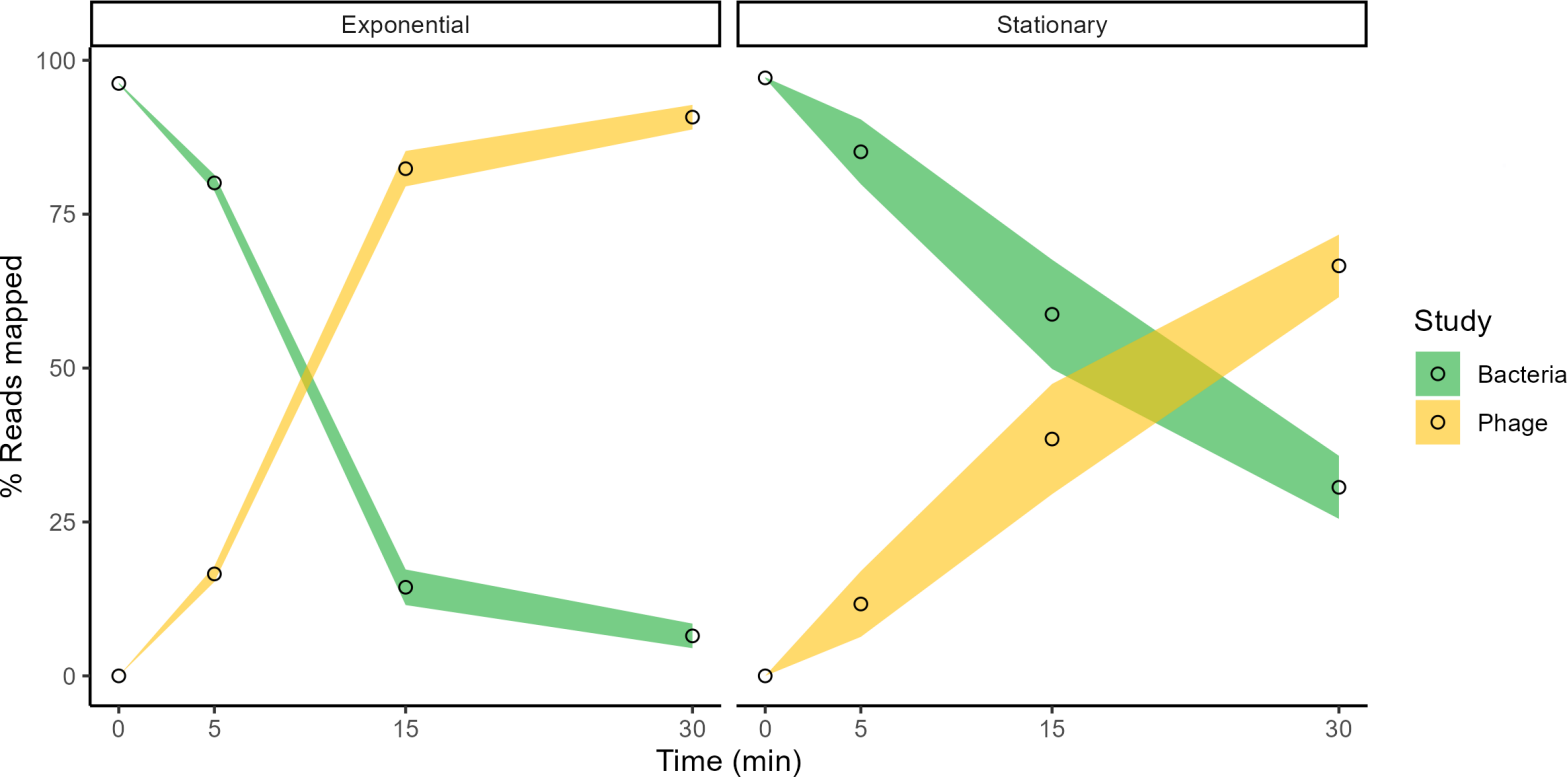
Percentage of reads that mapped to the genomes of *S. epidermidis* RP62A and SEP1 phage in exponential and stationary cultures during synchronized infections (MOI 5). Samples were collected before (0 min) and 5, 15, and 30 min after infection with SEP1. The values represent the mean ± standard deviation of three independent experiments.

RNA sequencing produced about 20 million reads for each sample, which were aligned to both bacterial and phage genomes. At time 0 (uninfected cultures), more than 96% of paired reads mapped to the *S. epidermidis* RP62A reference genome (Fig. 1). Five minutes post-infection, 14%–18% and 3%–21% of transcripts mapped to the SEP1 genome in exponential and stationary cultures, respectively. This percentage increased over time, with 88%–95% and 59%–76% of the respective total transcripts mapping to phage at 30 min. This indicates that SEP1 took over the transcriptional resources of both exponential and stationary cells.

A principal component analysis (PCA) of the reads that mapped to SEP1 showed that the different replicates from exponential cells were very similar at each time point, being very different between the three different time points. In stationary cells, this was not so clear, with the samples collected at different time points being less distinguishable from each other (Fig. S1).

**Fig 2:**
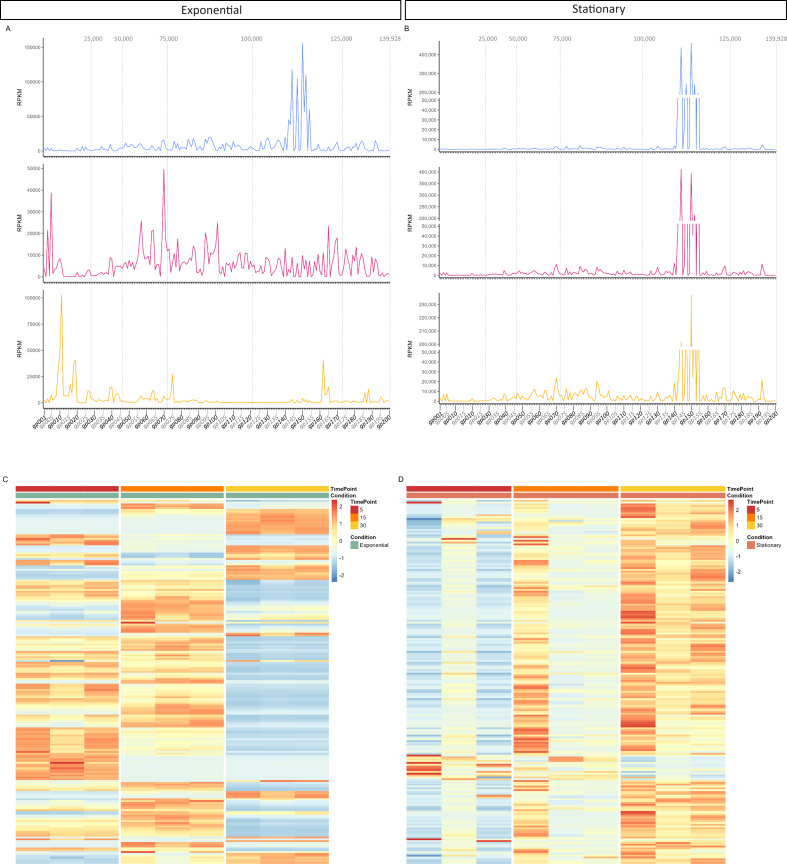
Transcriptional profile (**A and B**) and heatmap (**C and D**) of SEP1 phage genes at 5, 15, and 30 min post-infection in exponential (**A and C**) and stationary (**B and D**) cells. Represented are the RPKM mean values for each gene from three independent experiments.

Regarding the temporal transcriptional profiles of SEP1, a logical temporal expression can be observed in exponential cells (Fig. 2A). Based on the maximum RPKM (reads per kilobase per million mapped reads) mean values at 5, 15, and 30 min, 76 genes were classified as early genes, 78 as middle genes, and 46 as late genes (Fig. 2C; Table S1). At 5 min, a substantial expression of genes belonging to the module *gp142-gp154* was observed. This region contains genes encoding for long terminal repeat proteins, such as Trek (*gp144*), TreO (*gp145*), TreN-like membrane protein (*gp148*), and Tre (*gp151*), with the remaining genes codifying for hypothetical proteins. They have been reported to be involved in the host takeover, redirecting cell metabolism toward phage production (9). Middle genes with known functions are mostly involved in DNA replication and recombination and transcription regulation. Regarding late genes, most of them encode structural and assembly proteins, the lysis genes (endolysin and holin), as well as some hydrolases and the terminase small subunit (*gp200*). The hydrolases and the terminase are involved in phage packaging (10); therefore, their maximum expression at this late stage is expected. However, we verified a higher expression (although fold change [FC] < 2; *P*-value ≤ 0.05) of the terminase large subunit (*gp001*) at 5 min than at 15 and 30 min. This might be related to the presence of two introns fragmenting *gp001*, with the first intron encoding an HNH endonuclease (*gp002*). The presence of two group I introns within the large terminase gene is not exclusive of SEP1 and has been reported for other staphylococcal phages (11, 12).

In stationary cells, SEP1 transcription was delayed in comparison to what was observed in exponential cultures (Fig. 2B). Only *gp141–gp154* genes (except *gp143* and *gp153*) were more expressed, on average, at 5 min than at later time points (Fig. 2D; Table S1). A high transcription of some genes within the *gp142–gp154* module was observed, demonstrating that these should be the “very early” genes that are necessary to start the replication process. The *gp144*, *gp148*, *gp145,* and *gp152* of SEP1 are homologous to the *gp011*, *gp014*, *gp015,* and *gp018* of phage K. Indeed, in phage K-infected exponential cultures, high transcription of *gp014–gp018* genes from left and right long terminal repeats was observed already at 2 min, achieving their maximum within 10 min, leading to the hypothesis that they likely play a role in manipulating the phage genome inside the bacterium and establishing the phage replicating complex (13). Moreover, some of the genes within the *gp142–gp154* module were significantly more expressed in stationary- than exponential-infected cultures (Fig. S2). For instance, almost a fivefold difference was observed for *gp146*. Since transcription is delayed in stationary cells, we hypothesized that maximum transcription of these “very early” genes might have occurred before 5 min in exponential cells. However, by performing quantitative RT-PCR (qRT-PCR) with samples collected at 1, 2, and 5 min post-infection in exponential cells, we verified higher fold change at 5 min than at the earlier times (Fig. S3).

At 15 min, in addition to *gp143* and *gp153*, another 11 phage genes achieved their maximum average transcription in stationary-infected cultures. All of them encode hypothetical proteins, except the virion structural protein encoded by *gp025*. The remaining genes were transcribed at higher levels at the later time point analyzed (30 min).

Although the expression of early phage genes decreased over time in stationary cells, they remained constantly overexpressed compared to exponential cells. Even at 30 min post-infection, all genes between *gp141* and *gp154* (except *gp148*) were significantly more expressed in stationary-infected cells than in exponential cells, with fold changes ranging from 10 to 70 approximately. This could mean that at 30 min post-infection, SEP1 was still taking over the transcriptional resources of stationary cells, although the results show that infection has slightly progressed with the increase of transcription of the remaining genes. The elevated transcription levels may also result from the slower infection process, providing additional time for increased production of transcripts for these early genes.

### Host response to SEP1 infection

**Fig 3:**
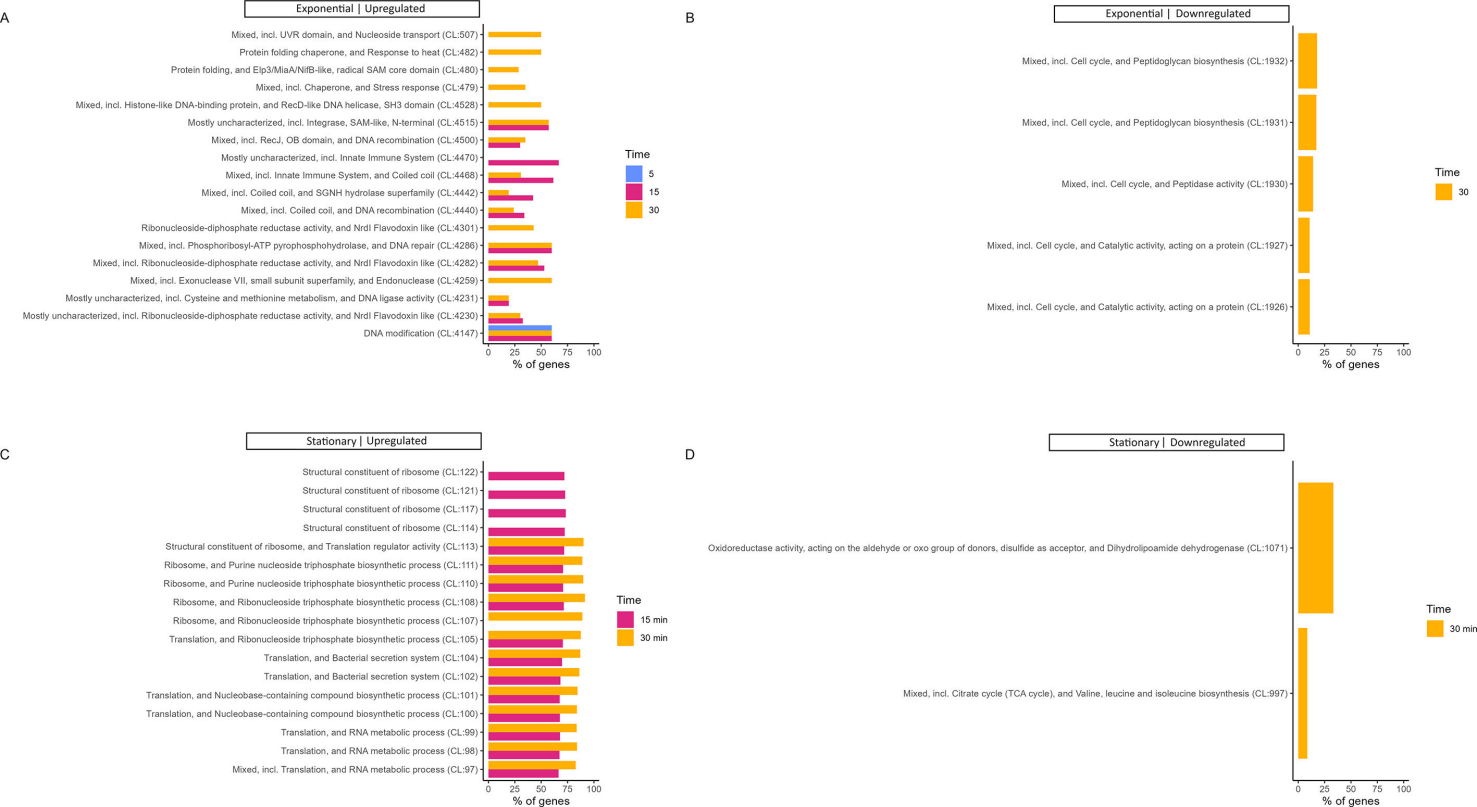
STRING functional enrichment analysis of upregulated (**A and C**) and downregulated (**B and D**) genes at 5, 15, and 30 min post-infection (comparing with time 0—uninfected control) in exponential (**A and B**) and stationary (**C and D**) cells. The values represent the percentage of genes that are differentially expressed within the enriched STRING cluster at different time points.

**Fig 4:**
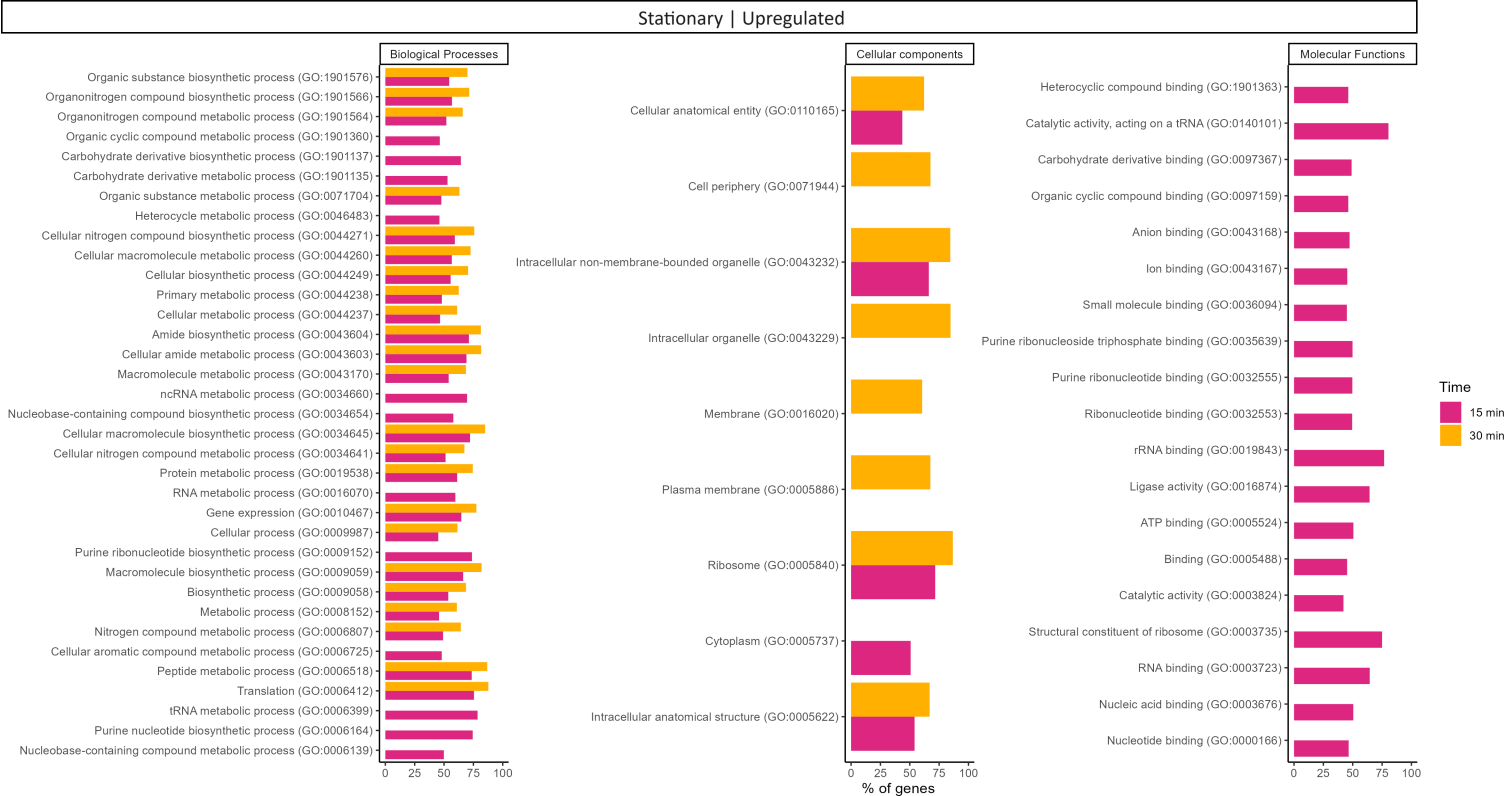
Gene ontology (GO) functional enrichment analysis (performed using STRING) of upregulated genes at 15 and 30 min post-infection (comparing with time 0—uninfected control) in stationary cells. The values represent the percentage of genes that are upregulated within the enriched GO cluster at different time points. No GO enrichments were obtained at 5 min post-infection in stationary cells or at 5, 15, and 30 min post-infection in exponential cells.

The response of the host to SEP1 infection over time was studied by analyzing the differentially expressed genes (DEGs) with FC ≥ 2 and a false discovery rate-adjusted *P*-value ≤ 0.05. The top 30 significantly more or less expressed genes in exponential- and stationary-infected cultures in comparison with their respective uninfected control in each time point were annotated (Table S2), and functional enrichment analysis of all DEGs was also performed (Fig. 3 and 4; Table S3).

At 5 min post-infection, exponential cells responded to phage only by upregulating three genes (*serp2473, hsdM*, and *serp2471*) that are involved in a type I DNA restriction-modification (R-M) system (Fig. 3; Table S2). R-M systems are bacterial defense mechanisms that interfere with the replication of the phage in the host. The host bacterium methylates its DNA recognition sites to distinguish it from unmodified foreign DNA, while the unmethylated phage DNA is cleaved. Type I R-M systems are composed of three subunits: *hsdS* (specificity), *hsdM* (methylation), and *hsdR* (restriction) (14–16). In this study, SERP2473 is a hypothetical protein (GenBank) but with a *hsdS* domain identified by Pfam (*E*-value = 1.2 × 10^−08^). The *serp2471* gene also encodes for the S subunit of the type I R-M system (two domains identified by Pfam with *E*-values = 2.4 × 10^−10^ and 4.3 × 10^−08^). The M subunit corresponds to the *hsdM* gene (*serp2472*). In exponential cells, these three genes were significantly upregulated already after 5 min of SEP1 infection, as stated before, increasing their expression even more at the 15 min time point (Table S2). In stationary cells, upregulation of these genes was not observed at 5 min, which agrees with the delayed SEP1 infection. At 15 min, however, *hsdM* and *serp2473* were about 92 and 66 times significantly more expressed than at time 0, increasing even more after 30 min, with the three genes being upregulated (Table S2). Despite this, upregulation of the *hsdR* gene was not observed for both exponential and stationary cells during the 30 min of infection. Therefore, we hypothesize that the host cells were not able to cleave the phage DNA, with phage bypassing the R-M system, as is evident from its successful replication. This may be explained by the fact that the phage was produced using the same bacterial strain used for the infection assays. Therefore, the phage DNA may have been methylated within the *S. epidermidis* cells, resulting in all progeny phages being methylated as well. In this scenario, the phage would be immune to the host’s R-M system, allowing infection to proceed without interference (17).

In exponential cells, 70 and 78 genes were, respectively, upregulated after 15 and 30 min of SEP1 infection, while in stationary cells, the number of positive DEGs was much higher, increasing from 29 at 5 min post-infection to 894 at 15 min, and 1,319 at 30 min. At 5 min post-infection in stationary cells (Table S2), some genes related to purine biosynthesis and ribosome biogenesis were already significantly more expressed, such as *purB* (adenylosuccinate lyase) and *rimM* (16S rRNA processing protein). After 15 and 30 min of SEP1 infection, STRING analysis showed an overrepresentation of genes involved in translation and RNA metabolic processes, particularly nucleobase-containing compound biosynthetic processes, and more specifically, ribonucleoside and purine biosynthetic processes, among other ribosomal-related clusters (Fig. 3C; Table S3). The functional analysis using STRING also showed that several gene ontology (GO) terms were significantly enriched (Fig. 4; Table S3). While at 15 min post-infection, 35 biological processes, 19 molecular functions, and 5 cellular components GO terms were enriched, at 30 min, only 23 biological processes and 8 cellular components GO terms were enriched. This analysis reinforced the observation that several genes involved in translation and several different metabolic and biosynthetic processes were significantly more expressed in stationary cells in response to SEP1 infection. It is also worth noting that, from the diverse molecular functions’ terms enriched at 15 min, 75% of genes that are structural constituents of the ribosome (GO:0003735) were upregulated. From the “cellular components” category, ribosome (GO:0005840) was the term with the highest percentage of genes, increasing from roughly 72% at 15 min to 87% at 30 min. Furthermore, Kyoto Encyclopedia of Genes and Genomes (KEGG) analysis also showed significant enrichment of the ribosome (ser03010) pathway at 15 min post-infection in stationary cells (Fig. S4; Table S3). Phages from the *Herelleviridae* family such as SEP1 do not encode their RNA polymerase, relying on the host RNA polymerase for their replication (10). The increase in transcription of the *S. epidermidis* RNA polymerase in stationary cells corroborates that SEP1 successfully hijacks the host transcription machinery, activating and redirecting stationary cells’ metabolism toward its replication. Indeed, at 15 min post-infection, the transcription of two subunits of the DNA-directed RNA polymerase, namely *rpoB* and *rpoC*, increased significantly. After 30 min of SEP1 infection, the FC of these genes was even higher. Also, *rpoA, rpoE, rpoZ,* and *rpoF* genes were significantly more expressed at 30 min. These results agree with our previous work in which, by qRT-PCR, we showed overexpression of the host RNA polymerase after phage addition in stationary cells, in contrast to exponential cells, where no significant changes in the RNA polymerase transcription were observed (6).

In exponential cultures, at 15 and 30 min post-infection, the functional enrichment analysis of upregulated genes (Fig. 3A; Table S3) revealed an overrepresentation of genes involved in DNA recombination, such as prophage genes. This included a recombinase from the phage integrase family (*serp1601*) and a DNA-binding protein HU (*serp1621*), upregulated at both time points, and a prophage holin (*xhlB*) and a lipase/acylhydrolase domain protein (*serp1649*), which were upregulated only at 15 min. A PHASTER analysis revealed that *S. epidermidis* RP62A contains one intact prophage (*Bacillus* phage SPBc2) and one incomplete prophage (*Bacillus* phage vB_BanS-Tsamsa) codifying 100 and 30 proteins, respectively. An in-depth analysis of genes within these regions, from *serp1500–serp1653*, revealed more upregulated putative phage-related proteins (Table S4). At 15 min, 15 upregulated genes are annotated in UniProt as “phage proteins” besides the *serp1518* gene encoding a “Siphovirus Gp157 family protein”; at 30 min, 14 “phage proteins” plus *serp1518* were upregulated, but only 10 of these genes are common to the two time points. Another 20 and 13 genes within this region were upregulated at 15 and 30 min post-infection, respectively, including another recombinase (*serp1501*). Only *serp1652* (phage protein) and *serp1586* (acetyltransferase) were downregulated 15 and 30 min after infection, respectively. The upregulation of prophage genes during virulent phage infection is a common feature observed in *Staphylococcus* and other bacterial genera as a response to stress (10, 13, 18, 19). The fact that not all prophage genes were upregulated indicates that the prophage was unable to complete its replication before the bacterium was lysed by SEP1. The functional enrichment analysis (Fig. 3A; Table S3) also highlighted other upregulated genes encoding, for instance, proteins with ribonucleoside-diphosphate reductase activity (NrdF-2, SERP1511, and SERP1513), thioredoxin (e.g., SERP1508), and DNA binding (e.g., SERP1502) and ligase (e.g., SERP1534) activity, which are also located within the prophage’s regions. Of note are also the anti-restriction proteins SERP1609, upregulated at 15 min, and SERP1608, upregulated at 15 and 30 min post-infection.

Similarly to what was observed in exponential cells, in stationary cells, some phage-related proteins were upregulated at 15 and 30 min post-infection (Table S4). A total of 15 and 42 genes located in the prophage’s region were, respectively, upregulated at these time points. These included 5 “phage proteins” and 3 “IS431mec-like transposases” at 15 min, and 14 “phage proteins” and the recombinase from the phage integrase family SERP1601 at 30 min.

After 30 min of exponential cells’ infection with SEP1, sets of genes involving chaperones and stress-response proteins, specifically heat response, were also overrepresented (Fig. 3A; Table S3). These genes are reported to participate in the refolding or the degradation of misfolded or defective proteins (20). Of these groups, the most significantly upregulated gene was the ATP-dependent Clp protease *clpB*, with an FC of 5.4. Also, the heat shock protein GrpE and the chaperone protein DnaK were about 2.4 times more expressed than at time 0. The genes *serp0163*, encoding a UvrB/UvrC domain protein involved in DNA repair, and *serp0164*, encoding the ATP:guanido phosphotransferase family protein McsB, were also significantly upregulated. However, a higher expression of the heat-inducible transcription repressor *hrcA* and the transcriptional regulator *ctsR*, which is a negative regulator of *clpB* transcription, was also observed. Similarly, heat shock response genes, such as *dnaK, clpB,* and *grpE*, were found to be upregulated in *Escherichia coli* challenged with PRD1 and φX174 phages at late infection times (21, 22). The authors hypothesized that the phage may benefit from their expression since they can help in the folding of phage proteins (22). In contrast, in *Staphylococcus aureus* biofilms infected with a sub-inhibitory dose of phiIPLA-RODI phage, a downregulation of heat shock response genes, including *clpB, dnaK,* and *grpE*, was observed (20).

Looking at the top 30 upregulated genes in exponential cells (Table S2), it is also worth mentioning *serp2119*, which encodes a conserved hypothetical protein with a putative YozE_SAM_like domain. This gene was about 7 and 14 times more expressed at 15 and 30 min post-infection, respectively. Its function is likely to be related to DNA binding (23). Importantly, the expression of *serp0224* (*vraX*) was increased by about five- and sevenfold after 15 and 30 min of SEP1 infection, respectively. In *S. aureus, vraX* expression is often observed as a stress response after exposure to antimicrobial agents, and it has been shown that it specifically inhibits the classical pathway of the complement system (24). In stationary cells, it is important to highlight *serp0048*, whose expression was increased by more than 150 times in stationary cells at 30 min post-infection, being the most upregulated gene (Table S2). It encodes a conserved hypothetical protein, and among the different motifs predicted by Pfam is the virulence factor BrkB, known to be responsible for resistance to complement-dependent killing by serum in *Bordetella pertussis* (25). This may contribute to the reduced efficacy of staphylococci phages in serum or plasma (26). The third most upregulated gene in stationary cells infected with SEP1 for 30 min is *serp0030*, which encodes a conserved hypothetical protein with a putative YozE_SAM_like domain, which, like previously discussed for *serp2119* in exponential cells, is probably involved in DNA binding.

While 94 and 109 genes were less expressed in exponential cells at 15 and 30 min post-infection in comparison with the uninfected control, only 1, 3, and 28 genes were found to be downregulated after 5, 15, and 30 min of SEP1 infection in stationary cells. The functional enrichment analysis in exponential cells at 30 min post-infection highlighted genes involved in the regulation of cell cycle and catalytic activity, particularly with peptidase activity, and, more specifically, in the synthesis of peptidoglycan (Fig. 3B; Table S3). In stationary cells, genes with acetoin dehydrogenase activity, an oxidoreductase, and an alcohol dehydrogenase, which are involved in the citrate cycle, and valine, leucine, and isoleucine biosynthesis are the ones highlighted (Fig. 3D; Table S3). Moreover, three KEGG pathways were found to be significantly enriched, specifically microbial metabolism in diverse environments, carbon metabolism, and glycolysis/glycogenesis (Fig. S4; Table S3).

A comparative analysis of differentially expressed genes between stationary and exponential cells at each time point was also performed to elucidate the temporal dynamics of host response to phage infection. Notably, significant differences in gene expression profiles between the two cell states were observed, with 96 genes being upregulated and 1,634 genes being downregulated at time 0 in stationary vs exponential cells. The number of upregulated genes increased over time, with 119, 168, and 171 genes being significantly more expressed in stationary cells than in exponential cells at 5, 15, and 30 min post-infection, respectively. Regarding downregulated genes, their number decreased from 1,262 at 5 min to 754 at 15 min and 329 at 30 min post-infection in stationary vs exponential cultures. For instance, at 5 min, only 35 out of the 119 upregulated genes were not upregulated as well at 0 min. A functional enrichment analysis of this group of genes only revealed an overrepresentation of genes involved in threonine biosynthesis. Concerning downregulated genes, only 25 out of 1,262, which were downregulated at 5 min, were not downregulated at the 0 time point, and no significant enrichment was obtained. Due to the inherent differences in cellular physiology between exponential and stationary cells, we consider that their direct comparison is not correct, making it difficult to draw any conclusions from these results. We could indeed compare the cells at the same infection stage, for instance, exponential cells at 5 min with stationary cells at a later time point, but we cannot be sure they are really in the same infection stage considering that the cells are really different from each other. We believe that analyzing the temporal dynamics of gene expression within each cell state offers valuable insights into the intricate interplay between phage and host.

In conclusion, we have shown that SEP1 effectively infects and replicates in both exponential and stationary cells, although slower in the former condition, and that the host employed a type I R-M system to defend against SEP1 infection. Most importantly, it was proved that SEP1 can activate diverse metabolic and biosynthetic processes in stationary cells. SEP1-like phages could be used not only as a better phage treatment approach but also to combat antibiotic tolerance by “awakening” cells.

Further proteomic studies should be performed to evaluate if changes in the proteome correlate well with the observed changes in the transcriptome of *S. epidermidis* exponential and stationary cells in response to SEP1 infection.

## MATERIALS AND METHODS

### Bacterial strains and culture conditions

The reference strain *S. epidermidis* 9142 (DSM No. 18857) was used in this study. Bacteria were grown in tryptic soy broth (TSB; VWR), tryptic soy agar (TSA; TSB with 1.2% [wt/vol] agar [LabKem]), or in TSA soft overlays (TSB with 0.3% [wt/vol] agar) at 37°C. Phage SEP1, specific for *S. epidermidis,* was previously isolated and characterized (6, 27).

### Phage production

SEP1 phage particles were produced using the double-agar-layer method as described before (6, 27). Briefly, 20 µL of phage suspension was spread on *S. epidermidis* 9142 lawns using paper strips. After 16 h of incubation at 37°C, 3 mL of SM buffer (100 mM NaCl, 8 mM MgSO_4_, 50 mM Tris/HCl [pH 7.5], 0.002% [wt/vol] gelatin) was added to each plate. The plates were agitated at 120 rpm in an orbital shaker (BIOSAN PSU-10i, Riga, Latvia) for 24 h at 4°C. Subsequently, the liquid and top agar were collected and centrifuged for 10 min, 9,000 × *g* at 4°C, and the supernatant was recovered. The phage concentration was determined as described previously (28). Samples were stored at 4°C until further use.

### Infection of planktonic cells

The infection of planktonic cells in both exponential and stationary phases was performed similarly to as described before (6). Briefly, to infect cells in the exponential phase, 200 µL of an overnight-grown culture of *S. epidermidis* 9142 was used to inoculate 20 mL of fresh TSB, and bacteria were allowed to grow at 37°C and 120 rpm, until an OD_600_ of approximately 0.35. Cells were infected with phage SEP1 at an MOI of 5 to ensure the synchronicity of the infection (95% of the bacterial population killed after 5 min phage-bacteria incubation). To induce the infection of stationary phase cells, *S. epidermidis* 9142 was grown for 48 h at 37°C and 120 rpm. Thereafter, part of the culture was centrifuged (9,000 × *g*, 10 min, 4°C), and the supernatant was used to dilute the remaining cell suspension to an OD_600_ of approximately 0.35. Stationary cells were then infected with phage SEP1 at the same MOI as exponential cells.

### Total RNA extraction and RNA-seq

Samples for RNA isolation were taken from the non-infected cultures shortly before infection and from the infected cultures at 5, 15, and 30 min post-infection.

Samples were immediately mixed with a 1:10 volume of an ice-cold stop solution (10% [vol/vol] buffered phenol and 90% [vol/vol] absolute ethanol) and kept on ice to stabilize RNA. Total RNA extraction was performed using the RNeasy Mini Kit (Qiagen) following the manufacturer’s instructions with some modifications. Briefly, samples were centrifuged (7,000 *g*, 5 min, 4°C), suspended in 600 µL of buffer RLT, and transferred to 2.0 mL tubes containing 0.1 mm silica beads (BeadBug prefilled tubes, Merck). Cells were lysed using the FastPrep cell disruptor (BIO 101, Thermo Scientific) at 6.5 m/s for 35 s. This step was repeated twice, with the samples being cooled on ice for 5 min between cycles. Afterward, the samples were centrifuged (5,000 × *g*, 5 min, 4°C), and the supernatants were transferred to a new tube and mixed with an equal volume of 70% ethanol. The suspensions were transferred to the RNeasy Mini spin columns, and the kit manufacturer’s instructions were followed, increasing the centrifugation time from 15 s to 1 min at 10,000 × *g* (room temperature). RNA was eluted in 40 µL of RNase-free water. To degrade genomic DNA, the TURBO DNA-free Kit (Thermo Scientific) was used. RNA concentration and purity were determined using a NanoDrop 1000 spectrophotometer (Thermo Scientific), and RNA integrity was assessed by visualization of the 23S/16S banding pattern in non-denaturing 1% agarose gel electrophoresis. RNA integrity was then determined using the Bioanalyzer 2100 equipment and an RNA 6000 Pico Kit (Agilent Technologies). Samples from three independent experiments with an RNA Integrity Number above 8 were selected for RNA sequencing. RNA samples were sent to Novogene (UK) for library preparation. Briefly, ribosomal RNA was removed from total RNA, followed by ethanol precipitation. After fragmentation, the first strand of cDNA was synthesized using random hexamer primers. During the second strand cDNA synthesis, dUTPs were replaced with dTTPs in the reaction buffer. The directional library was ready after end repair, A-tailing, adapter ligation, size selection, USER enzyme digestion, amplification, and purification. The library was then checked with Qubit and qRT-PCR for quantification and bioanalyzer for size distribution detection. Quantified libraries were pooled and sequenced using the Illumina NovaSeq 6000 Sequencing System (paired end 150 bp), according to library concentration and data amount required. The quality scores of the sequencing data are provided in the supplemental material (Table S5.1).

### RNA-seq data processing, functional annotation, enrichment analysis, and statistical analysis

CLC Genomics Workbench version 21 (Qiagen) was used for sequence quality trimming and mapping to reference genomes, which were performed following default parameters (Tables S5.2 and S5.3), and normalization of gene expression. Trimmed reads were aligned to the reference genome of *S. epidermidis* RP62A (GenBank accession number CP000029) and the genome of SEP1 (GenBank accession number NC_041928.1) using the build-in CLC read mapper. In Table S5.4, a summary of the reports obtained in quality trimming and mapping to either bacteria or phage genomes can be seen. Gene expression was normalized by calculating RPKM. Baggerley’s test was applied to identify statistically significant alterations between different time points (0 min vs 5, 15, and 30 min post-infection) and cell state (exponential vs stationary cells). Alterations with fold changes below two and *P*-values above 0.05 were discarded.

Gene function was annotated primarily based on the Search Tool for the Retrieval of Interacting Genes/Proteins (STRING, version 12.0) (29) and Kyoto Encyclopedia of Genes and Genomes (KEGG) (30). For some hypothetical proteins, the BLAST (31) tool and the Pfam (32) and UniProt (33) databases were also consulted to investigate their putative function.

Functional enrichment analysis of differentially expressed genes was performed using STRING (which includes gene ontology terms, KEGG pathways, and STRING local neighborhood clusters) with default parameters (medium confidence [0.400]; medium [5%] false discovery rate stringency).

The Phaster tool was used for analyzing the presence of prophage regions within the genome of *S. epidermidis* RP62A (34).

The data generated were analyzed in R version 4.2.3 (https://cran.r-project.org/) embedded in R Studio 2023.03.0 Build 386 (https://www.rstudio.com/) using the R package ggplot2 to generate the plots. Package pheatmap was used to generate heatmap plots and factoextra to generate PCA plots.

### Quantitative RT-PCR

For quantitative RT-PCR analysis, RNA was extracted from exponential cells shortly before infection and from SEP1-infected cultures (MOI 5) at 1, 2, and 5 min post-infection, as described previously. Genomic DNA was degraded using the TURBO DNA-free Kit (Thermo Scientific), and RNA concentration and purity were determined using a NanoDrop 1000 spectrophotometer (Thermo Scientific). Total RNA samples were reverse transcribed using Xpert cDNA Synthesis Kit (GRISP), following the manufacturer’s instructions. Control reactions lacking the reverse transcriptase enzyme (no-RT) were included. qPCR was used to assess the expression of SEP1 phage *gp146* (primer forward: 5′ TTGTTACAAATGAGAGAATTAGAAC 3′; primer reverse: 5′ TTATTTTTGTAACTCTTCTAGTTC 3′). The 16S rRNA gene of *S. epidermidis* 9142 was used as a reference gene (primer forward: 5′ GGGCTACACACGTGCTACAA 3′; primer reverse: 5′ GTACAAGACCCGGGAACGTA 3′). Reactions contained 2 µL of 1:100 diluted cDNA or no-RT control, 0.5 µL of each primer (10 pmol/µL), and 5 µL Xpert Fast SYBR 2× (GRISP), with the following thermal cycler parameters: 95°C for 3 min, 40 cycles of 95°C for 5 s, and 60°C for 30 s. To monitor the reaction specificity and primer dimer formation, end products were analyzed by melting curves. A no-template control lacking the cDNA template was also performed. The relative quantification of gene expression was determined using the Pfaffl method (35). Data analysis was based on three independent experiments.

## Data Availability

Raw reads and processed data sets have been deposited in NCBI’s Gene Expression Omnibus (36) and are accessible through GEO Series accession number GSE254200. Analysis codes and processed data sets are also publicly available in the GitHub repository: https://github.com/GracaP88/Phage_Stationary.

## SUPPLEMENTAL MATERIAL

Fig. S1 msystems.00263-24-s0001.tiff Principal component analysis graph.

Fig. S2 msystems.00263-24-s0002.tiff Heatmap of gp142 to gp154 SEP1 phage genes.

Fig. S3 msystems.00263-24-s0003.tif *S. epidermidis* 9142 exponential cell gene expression (qPCR).

Fig. S4 msystems.00263-24-s0004.tif KEGG functional enrichment analysis of upregulated and downregulated genes at 30 min post-infection in stationary cells.

Supplemental tables msystems.00263-24-s0005.xlsx Tables S1 to S5.

ASM does not own the copyrights to Supplemental Material that may be linked to, or accessed through, an article. The authors have granted ASM a non-exclusive, world-wide license to publish the Supplemental Material files. Please contact the corresponding author directly for reuse.
