# Supplementary figures and images for: Phage SEP1 hijacks Staphylococcus epidermidis stationary cells’ metabolism to replicate
Source: mSystems. 2024 Jun 21;9(7):e00263-24. doi: 10.1128/msystems.00263-24 (PMC11265418; doi:10.1128/msystems.00263-24)

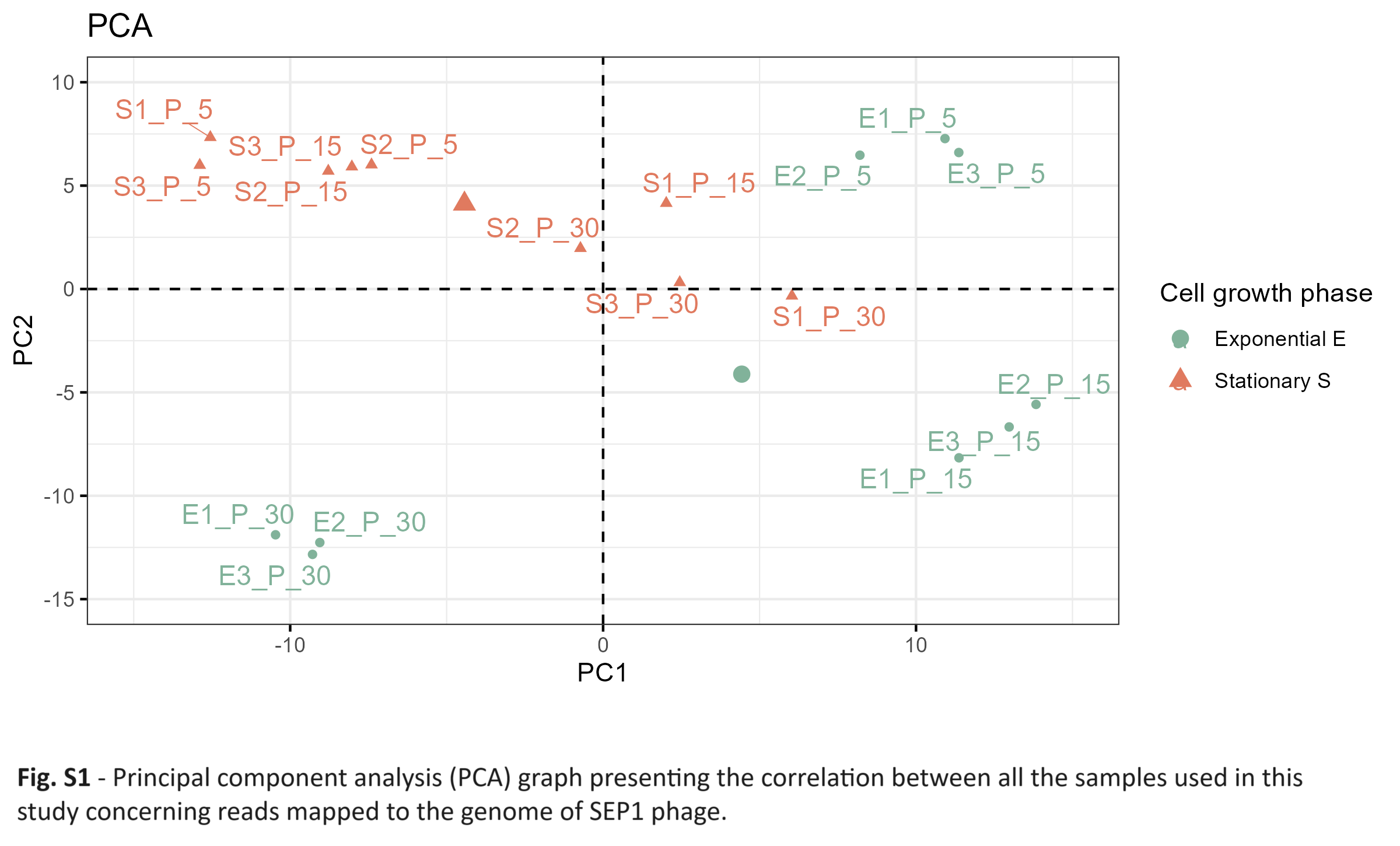

Supplement: Fig. S1 — Principal component analysis graph. [file msystems.00263-24-s0001.tiff]

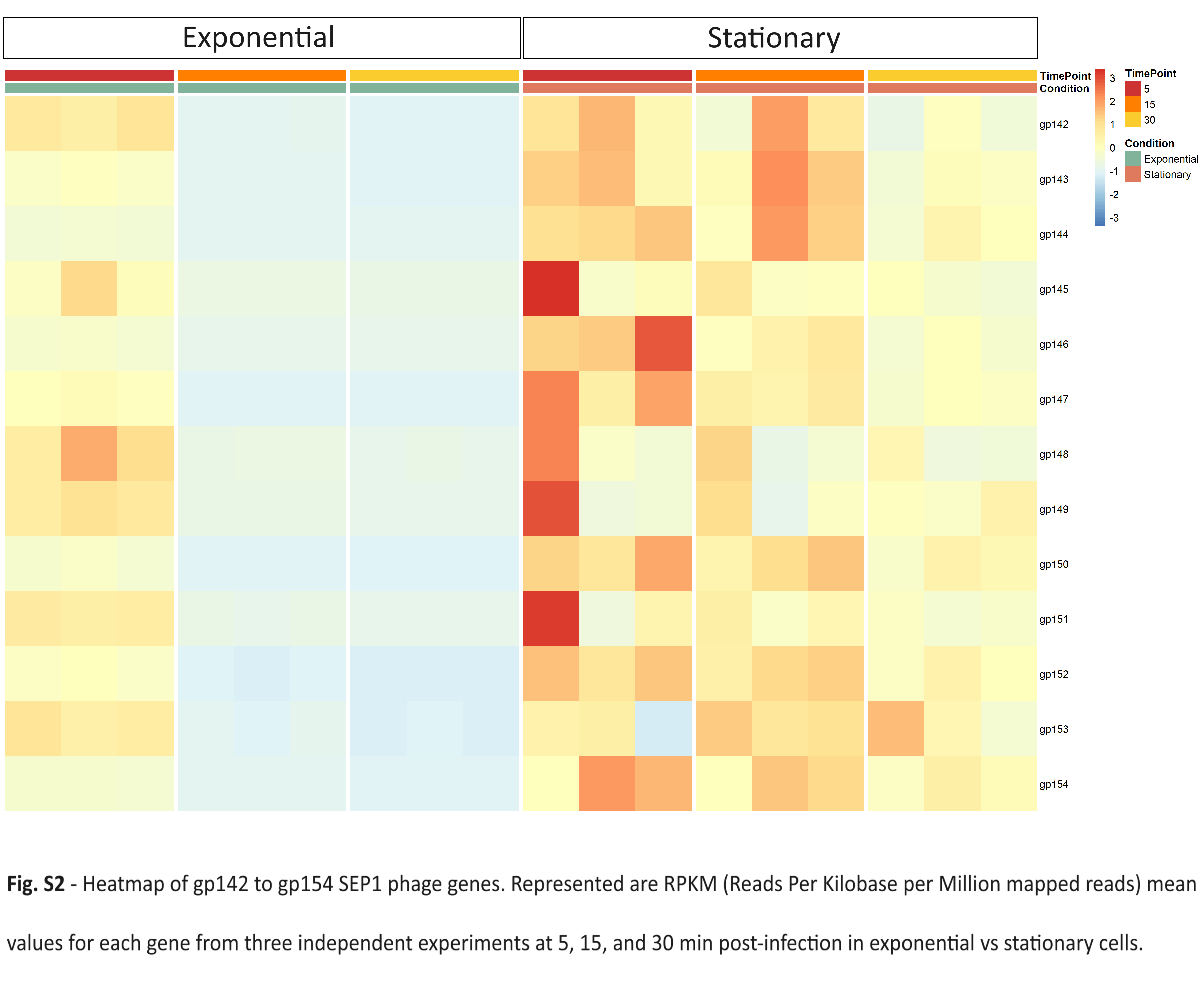

Supplement: Fig. S2 — Heatmap of gp142 to gp154 SEP1 phage genes. [file msystems.00263-24-s0002.tiff]

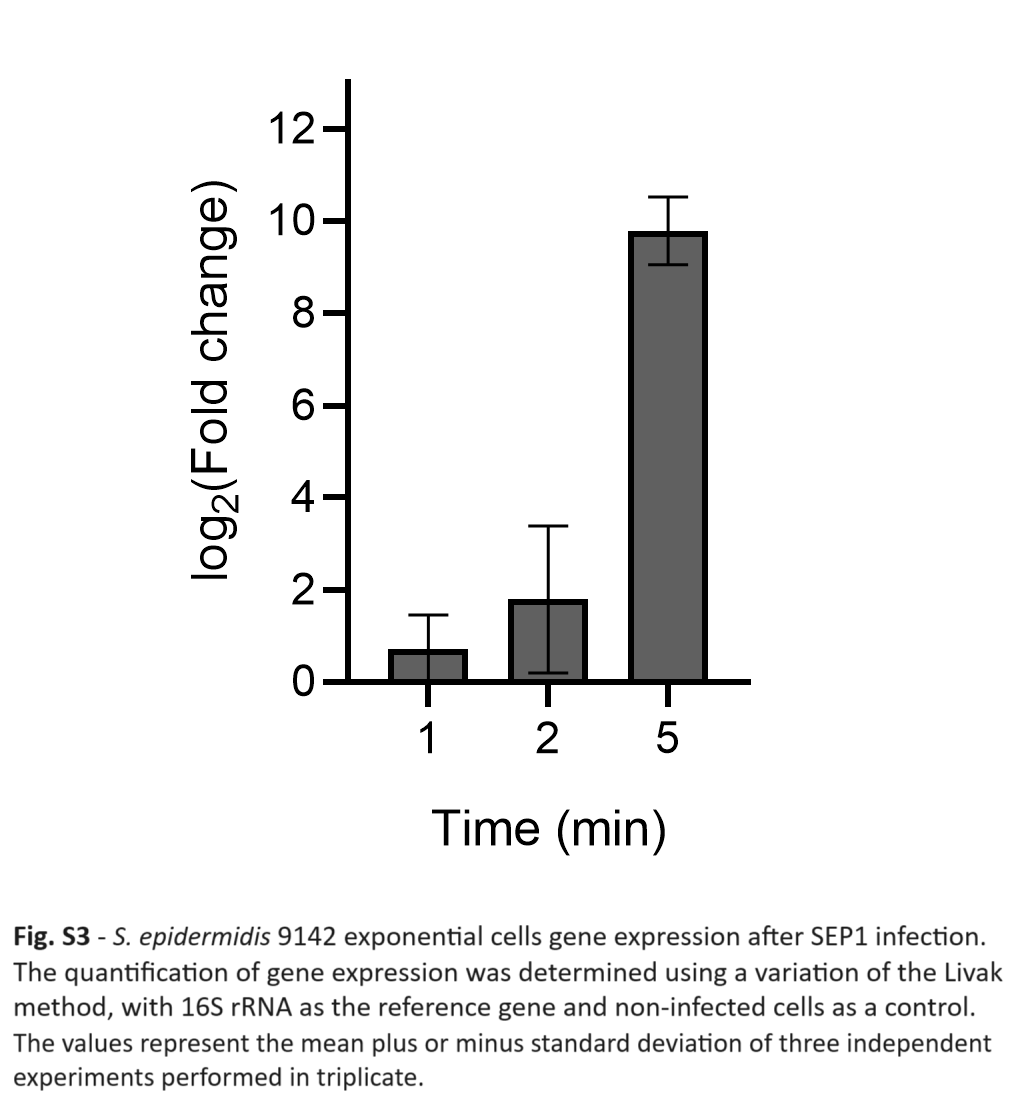

Supplement: Fig. S3 — S. epidermidis 9142 exponential cell gene expression (qPCR). [file msystems.00263-24-s0003.tif]

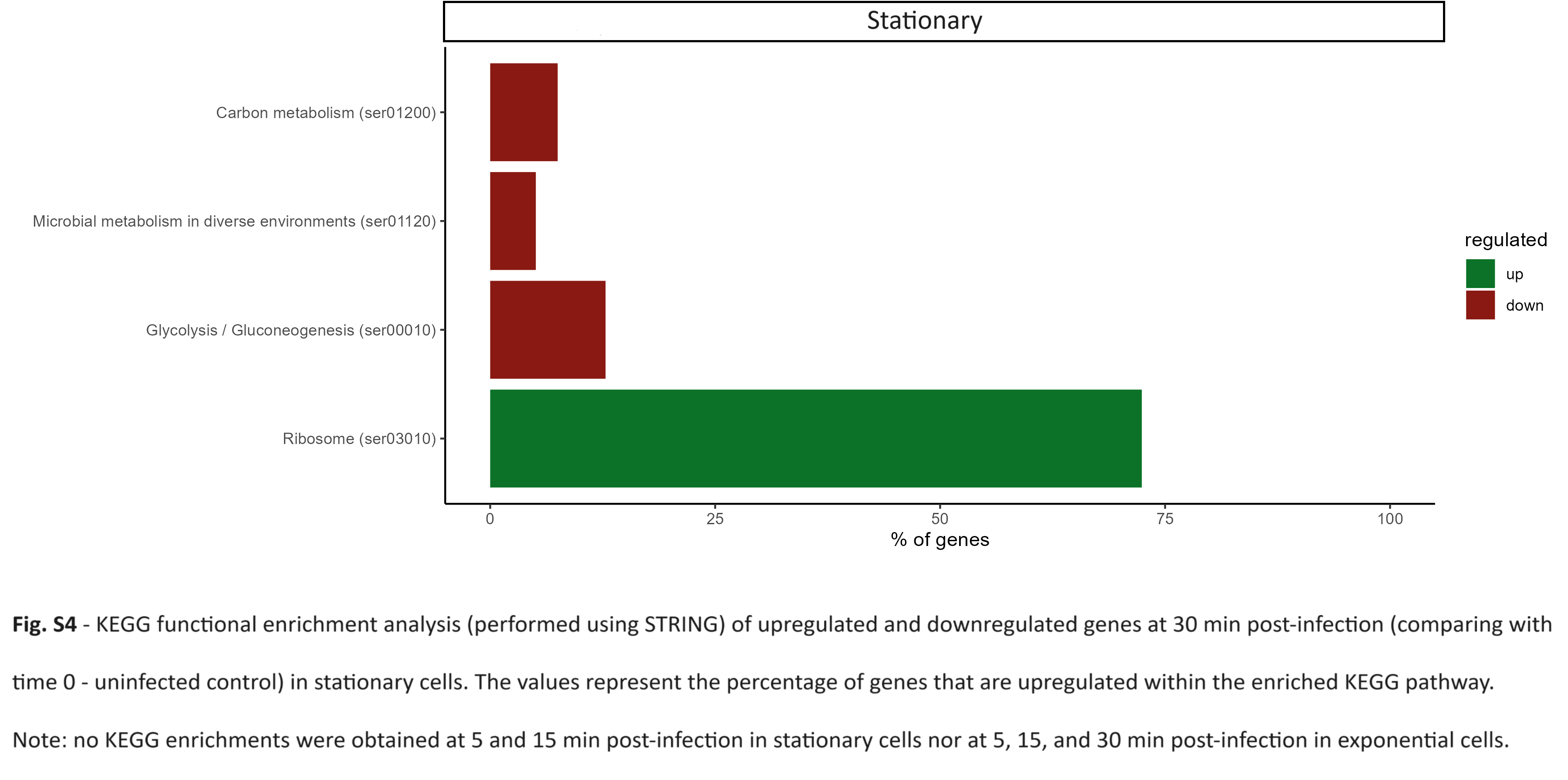

Supplement: Fig. S4 — KEGG functional enrichment analysis of upregulated and downregulated genes at 30 min post-infection in stationary cells. [file msystems.00263-24-s0004.tif]
